# Intracerebroventricular Neuropeptide FF Diminishes the Number of Apneas and Cardiovascular Effects Produced by Opioid Receptors’ Activation

**DOI:** 10.3390/ijms21238931

**Published:** 2020-11-25

**Authors:** Piotr Wojciechowski, Kryspin Andrzejewski, Katarzyna Kaczyńska

**Affiliations:** Department of Respiration Physiology, Mossakowski Medical Research Centre, Polish Academy of Sciences, 02-105 Warsaw, Poland; kandrzejewski@imdik.pan.pl

**Keywords:** neuropeptide FF, NPFF receptors, μ-opioid receptors, endomorphin-1, apnea

## Abstract

The opioid-induced analgesia is associated with a number of side effects such as addiction, tolerance and respiratory depression. The involvement of neuropeptide FF (NPFF) in modulation of pain perception, opioid-induced tolerance and dependence was well documented in contrast to respiratory depression. Therefore, the aim of the present study was to examine the potency of NPFF to block post-opioid respiratory depression, one of the main adverse effects of opioid therapy. Urethane-chloralose anaesthetized Wistar rats were injected either intravenously (iv) or intracerebroventricularly (icv) with various doses of NPFF prior to iv endomorphin-1 (EM-1) administration. Iv NPFF diminished the number of EM-1-induced apneas without affecting their length and without influence on the EM-1 induced blood pressure decline. Icv pretreatment with NPFF abolished the occurrence of post-EM-1 apneas and reduced also the maximal drop in blood pressure and heart rate. These effects were completely blocked by the NPFF receptor antagonist RF9, which was given as a mixture with NPFF before systemic EM-1 administration. In conclusion, our results showed that centrally administered neuropeptide FF is effective in preventing apnea evoked by stimulation of μ-opioid receptors and the effect was due to activation of central NPFF receptors. Our finding indicates a potential target for reversal of opioid-induced respiratory depression.

## 1. Introduction

The opioid-induced analgesia is associated with a number of side effects such as development of tolerance, addiction and respiratory depression. The activity of opioids undergoes regulation by homeostatic antiopioid systems involving various neuropeptides: neuropeptide FF (NPFF), cholecystokinin (CCK), Tyr-MIF-1-related peptide, nociceptin/orphanin FQ and several others [1].

Neuropeptide FF (FLFQPQRFa) is a mammalian amidated neuropeptide originally isolated from bovine brain in 1985 and described as a pain modulating peptide with antiopioid activity on the morphine-induced analgesia in the rat [2].

The distribution of NPFF and its receptors in the central and peripheral nervous system [3,4,5] indicates their contribution to the modulation of various physiological functions including water balance [6,7], food consumption [8,9], neuroendocrine processes [10,11], intestinal motility [12,13] and body temperature [14].

A number of data indicates the involvement of NPFF system in cardiovascular regulation [15,16,17,18,19]. Intravenous administration of NPFF results in dose-dependent increase in blood pressure and heart rate [20,21,22], however microinjected into the rat NTS, to the contrast, it evokes bradycardia [23].

The most researched NPFF activity concerns its influence on opioid-induced analgesia. It has been shown that perception and processing of nociceptive stimuli and activity of opioids are modified by the NPFF system [24,25,26,27,28]. NPFF and its receptors were detected in dorsal spinal cord, and various nuclei of pons and medulla [3,29], the structures supported also with the presence of opioid receptors [30,31,32].

NPFF has no pronociceptive effects by itself and does not bind to opioid receptors [33] but in different way modulates opioid-induced antinociception, depending on the site of administration [26,27,34]. The intracerebroventricular (icv) or intrathecal injection of NPFF in rodents exhibits either antiopioid activities or opioid-like effects, respectively [27]. After icv administration, NPFF reverses morphine antinociception in the tail-flick test, both in the rat and mouse [2,26,34,35,36] and produces an abstinence syndrome in the naive and morphine-tolerant rat [5,37,38]. Intrathecal administration of NPFF potentiates morphine-induced analgesia [39]. NPFF analogues, F8F-, and FMRF amide, administered in the same route either enhance (F8F amide) or decrease (FMRF amide) spinal morphine analgesia [40].

Majority of data on the crosstalk between NPFF and opioid system concentrates on its effects on the opioid-induced analgesia, tolerance, dependence, or modulation of the reward system and associated behaviors. There is no information on how NPFF affects respiratory pattern by itself and whether this neuropeptide is able to reverse opioid-induced respiratory depression, one of the main adverse effects of opioid therapy. Therefore, we decided to investigate whether systemic or icv administration of NPFF affects post-opioid arrest of breathing and whether NPFF receptors are involved in this effect.

## 2. Results

### 2.1. The Effects of Iv NPFF on Cardiovascular and Respiratory Pattern

**Figure 1:**
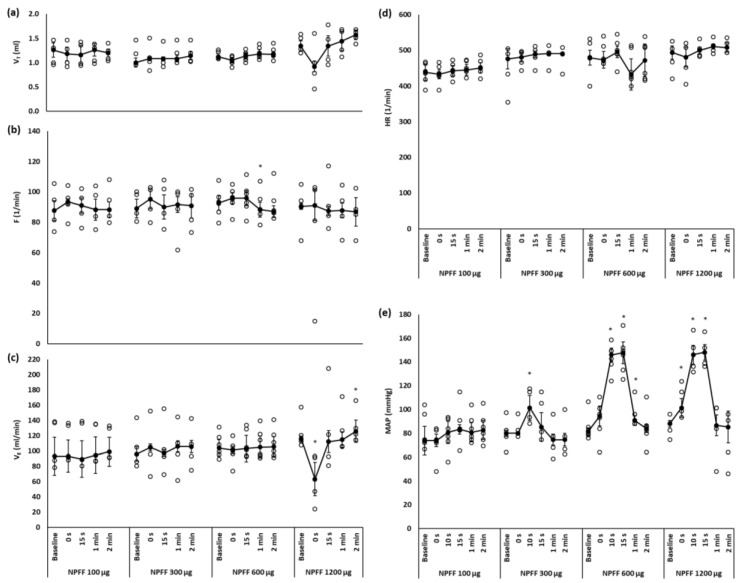
Changes over time of (**a**) tidal volume (V_T_); (**b**) frequency of breathing (F), (**c**) minute ventilation (V_E_), (**d**) heart rate (HR) and (**e**) mean arterial blood pressure (MAP) after iv injection of various doses of NPFF. Significant drop in V_E_ is produced by the highest dose of NPFF. Short-lived increase in MAP is evoked mostly by the two highest doses of NPFF. The data are presented as median ± QD; * *p* < 0.05 vs. baseline value; *n* = 5–6 per dose.

Saline injected intravenously (iv) or into the right cerebral ventricle (icv) had no effect on respiratory or cardiovascular variables. Figure 1 shows the effect of intravenous injection of NPFF on respiratory and cardiovascular variables. The doses tested were selected on the basis of a previous study that examined cardiovascular effects of intravenous NPFF [20]. Among the doses of NPFF we tested (0.1, 0.3, 0.6 and 1.2 mg/kg) only the highest one significantly affected the respiration. It caused immediate and short-lived, however insignificant drop in tidal volume, which transformed into the decrease in minute ventilation (*p* < 0.05). Unlike other doses of NPFF the highest one used in the present experiments evoked a single episode of apnea lasting 3.7 s.

NPFF injected intravenously did not affect heart rate (HR; Figure 1d) but evoked dose dependent increase in mean arterial blood pressure (MAP) with the hypertensive effect being marked the most after the doses of 0.6 and 1.2 mg/kg (Figure 1e). Due to influence on the respiratory pattern, the highest dose of NPFF was excluded from experiments testing its possibility to prevent post-opioid arrest of breathing.

### 2.2. The Effects of Iv NPFF on Apnea and Cardiovascular Effects Induced by EM-1

**Figure 2:**
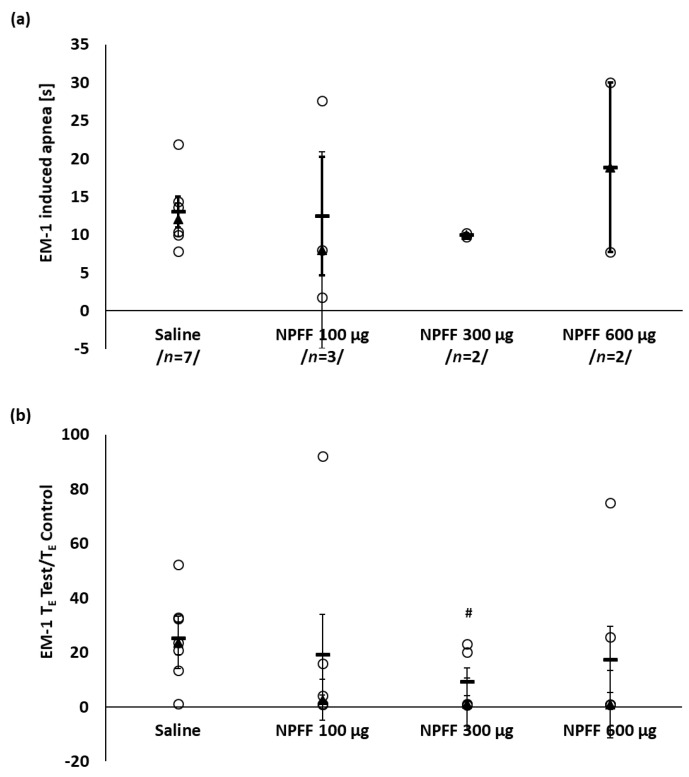
The effect of iv injected NPFF on the number and duration of EM-1-induced apneas (**a**) and T_E_ test/T_E_ control ratio (**b**). The data has been shown as median ± QD (▲, thin whiskers) and mean ± SEM (−, bold whiskers); ○ episode of apnea or T_E_ test/T_E_ control ratio of a single rat. The number of apnea episodes are shown in-between slashes (**a**). Mann–Whitney test: # *p* < 0.05 vs. control (saline) group. Total number of tested animals per each NPFF dose: *n* = 5–7.

Systemic injection of endomorphin 1 (EM-1) at a dose of 50 μg/kg, after saline pretreatment, evoked the apnea of median duration of 12 ± 2.2 s in all tested rats (Figure 2a). The dose of EM-1 was established in our previous experiments [41].

Figure 2a shows that intravenous pretreatment with various doses of NPFF did not prevent completely the occurrence of post-EM-1 apneas but there was a tendency to limit their number to 2 episodes per 5 and 6 animals pretreated with 0.3 or 0.6 mg/kg NPFF, respectively. The median duration of T_E_ test/T_E_ control ratio appeared to be significantly reduced after pretreatment with a dose of 0.3 mg/kg NPFF in comparison to control saline pretreated group (Figure 2b).

EM-1 control injection evoked significant slowdown in the heart rate from baseline median value of 473 ± 37 to 396 ± 18 mmHg (Table 1) and decline in MAP. During the first 30 s after EM-1 administration a drop from the initial 80 ± 6 mmHg to a median minimal value of 29 ± 11 mmHg (*p* < 0.05) was observed (Table 1). Iv NPFF was without effect on the post EM-1 hypotension at any of doses used (Table 1). The slowing of the heart rate was significantly reduced only with a dose of 0.6 mg/kg.

### 2.3. The Effects of Icv NPFF on Apnea and Cardiovascular Effects Induced by EM-1

**Figure 3:**
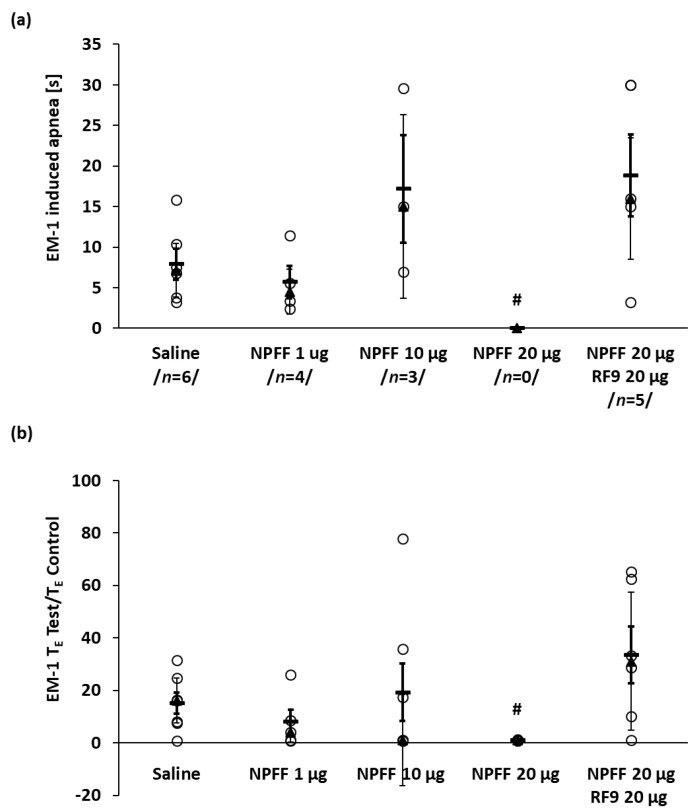
The dose dependent effect of icv NPFF administration on the number and duration of EM1-induced apneas (**a**) and T_E_ test/T_E_ control ratio (**b**). Note that NPFF at a dose of 20 μg eliminated EM-1 provoked apnea completely. The effect was blocked by NPFF receptor antagonist RF9. The data has been shown as median ± QD (▲, thin whiskers) and mean ± SEM (**−**, bold whiskers); ○ episode of apnea or T_E_ test/T_E_ control ratio of a single rat. The number of apnea episodes are shown in-between slashes (**a**). Mann–Whitney test: # *p* < 0.05 vs. control (saline) group. Total number of examined animals per each NPFF dose: *n* = 5–7.

**Figure 4:**
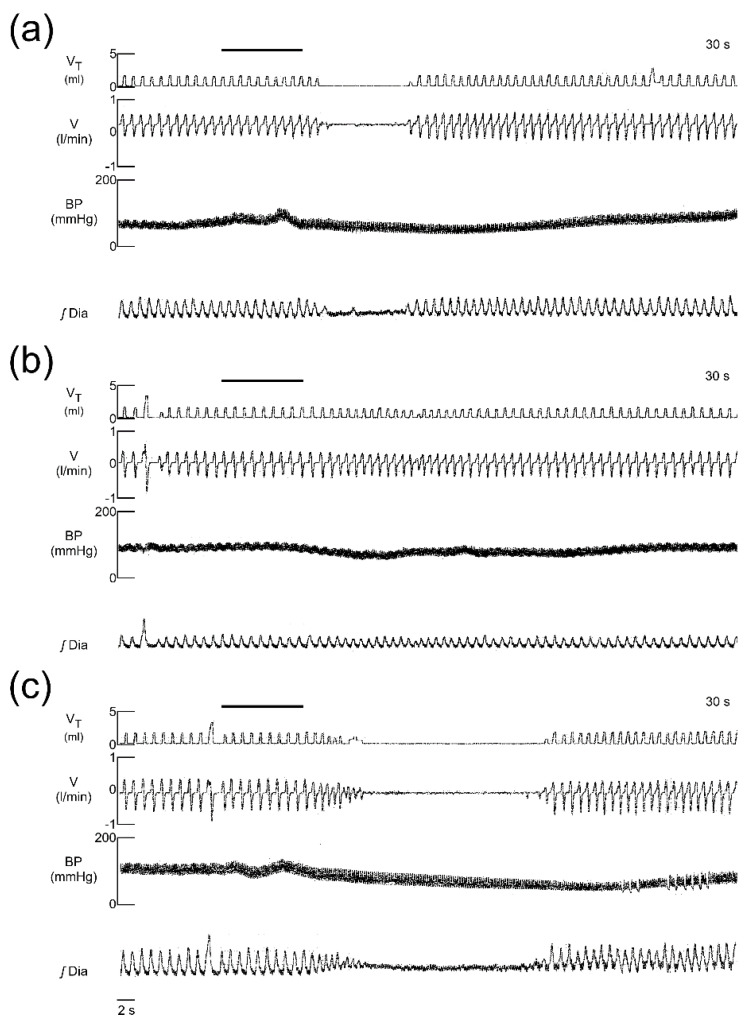
Representative recordings of respiratory and blood pressure responses to intravenous EM-1 injection in: control rat injected icv with saline (**a**); pretreated with icv injection of 20 μg NPFF (**b**); and pretreated with icv injection of a mixture of 20 μg NPFF and 20 μg RF9 (an antagonist of NPFF receptors). The line above top traces shows the injection of EM-1. Appearance of apnea and a drop in arterial blood pressure after EM-1 injection (**a**); icv injection of NPFF abolished the presence of the apnea and hypotensive effect of EM-1 injection (**b**); coadministration of NPFF and RF9 restored the EM-1 ability to induce apnea and blood pressure decrease (**c**). V_T_—tidal volume; V—airflow; BP—blood pressure; ʃDIA—integrated electromyogram of the diaphragm.

As the systemic administration of neuropeptide FF was not particularly effective in blocking the cardiovascular and respiratory effects of EM-1 we decided to administer NPFF centrally. EM-1 control iv administration after icv saline injection evoked immediate arrest of breathing in 6 of 7 tested rats (Figure 3a and Figure 4a) and a significant decrease in MAP and heart rate (Table 2). The tested doses of NPFF we used were selected on the basis of earlier studies where NPFF was applied intracerebroventricularly [37,42,43]. NPFF was injected icv slowly for 5 min and remained without influence on cardiovascular and respiratory variables. The icv injection of the NPFF dose-dependently limited the number of EM-1-induced apneas (Figure 3a) and at the dose of 20 μg abolished its presence completely (Figure 3a and Figure 4b), reducing the T_E_ test/T_E_ control ratio from 16.2 ± 8.6 s in saline group to the 1.0 ± 0.1 s (Figure 3b). The latter dose and smaller one of 10 μg of this antiopioid peptide reduced also the maximal MAP and HR drop induced by EM-1 (Table 2).

### 2.4. The Effect of Blockade with RF9

The modulatory effect of NPFF on the presence and duration of the apnea, and heart rate decrease and hypotension were prevented by the NPFF receptors’ antagonist RF9 (Figure 3 and Table 2). Iv EM-1, despite icv pretreatment with a mixture of NPFF and RF9, evoked apnea in all rats of median duration 16 ± 7.5 s, and a significant drop in MAP from 73 ± 7 to 52 ± 16 mmHg (Figure 4) and slowdown in the heart rate from 413 ± 56 to 354 ± 33 beats/min (Table 2).

## 3. Discussion

Opioid treatments are associated with several side effects, including addiction, the development of tolerance, and life-threatening respiratory depression. NPFF is a neuromodulator showing antiopioid activity such as counteracting morphine-induced tolerance [36], analgesia [27,34], and rewarding effect [44]. The present study is the first that investigated respiratory effects of intravenously administered NPFF and its potency to reverse respiratory depression induced by activation of μ-opioid receptors. The main finding of our study is that NPFF injected iv reduces the number of respiratory arrests while after the icv challenge the neuropeptide FF prevents completely apnea presence induced by endomorphin1 administration. Furthermore, NPFF in the tested doses used to pretreat EM-1 injection did not produce significant changes in the respiratory pattern. The exception was the highest iv dose of 1.2 mg/kg that evoked short-lived depression of minute ventilation and single episode of apnea in one rat. Therefore, this dose was not used to pretreat μ-opioid receptors stimulation by EM-1. Nevertheless, it is puzzling by what mechanism NPFF exerts its effect on respiration at the highest dose or how other doses were able to reduce apnea number although not its average longevity after the systemic challenge.

NPFF most probably does not penetrate the blood brain barrier (BBB) [20]. There is also no information on the presence of NPFF receptors on vagal nerves, which could be responsible for the effect. Therefore we suppose that highest doses could reach to the permeable circumventricular organ in the region of area postrema such as the nucleus of the tractus solitarii (NTS) [45] and there exerts its effect on respiration. This sensory nucleus of dorsomedial medulla oblongata is the primary center integrating information from cardiorespiratory viscera participating in cardiovascular and respiratory control [46,47]. It receives sensory inputs from lung, arterial, and carotid body receptors via the vagus and glossopharyngeal nerve fibers, respectively, and regulates respiratory circuits through its projections to areas involved in respiratory rhythm generation and formation of the respiratory pattern [45,48].

Our study showed that central NPFF injection was much more effective in preventing post-EM-1 apnea than systemic administration. The episodes of apnea induced with EM-1 intravenous application were previously described as the result of vagal μ-opioid receptors stimulation [41].

Mechanism of apnea prevention by NPFF icv administration is unknown. We do not have direct evidence but can speculate that the effect could be mediated via acting on NTS NPFF receptors. NTS is one of the main locations of NPFF-immunopositive cell bodies and NPFF receptors [23,29,49]. In 2002 Jhamandas and McTavish [50] have demonstrated that icv injected NPFF was able to increase the number of activated neurons and gene expression of NPFF in the NTS. Therefore, it is plausible that also in our study NPFF via diffusion from cerebral ventricle could have a direct effect on NTS neurons supplied with NPFF receptors. Acting on its receptors located in the NTS NPFF could inhibit EM-1 produced and vagally mediated arrest of breathing [41]. This supposition is supported by conviction that NTS is a central link between vagal sensory afferents and respiratory bulbopontine structures and a principal relay station for reflex apnea [51]. Zhuang et al., 2017 [52] showed previously that microinjection of the μ-opioid receptor agonist into caudomedial NTS blocked bronchopulmonary C-fiber mediated apnea. The latter was induced by intraatrial capsaicin administration and although NTS synaptic processing of bronchopulmonary C-fiber apnea activated by stimulation of vanilloid and μ-opioid can be different, the study showed that μ-opioid receptors of caudomedial NTS are critical for respiratory responses to excitation of vagal C-fibers. In the present study it is plausible that stimulation of central NPFF receptors modulated central opioid system and prevented vagally mediated opioid-induced apnea. Although the mechanism of the interaction between opioid and NPFF systems is unclear, coexistence of abundantly expressed μ-opioid receptors in the NTS [52,53,54] with NPFF receptors [29] suggest possible interaction. Interestingly, the density of μ-opioid-binding sites in brain was confirmed to be regulated via NPFF system because immunoneutralization of NPFF led to its increase [55]. Considering the opposite situation, we can speculate that activation of NPFF receptors in the NTS could via unknown mechanism inhibit or downregulate opioid receptors and prevent apnea appearance. In the similar manner to our study it was also previously shown that central antinociception produced by EM-1 can be reduced by icv pretreatment with NPFF [43].

Nevertheless, further experiments are needed with a more selective site of NPFF injection such as NTS to show the exact location of NPFF action on opioid-induced apnea. The involvement of NPFF receptors was reinforced by our finding that central coadministration of NPFF and its antagonist was able to prevent the blocking effect of NPFF on EM-1 induced apneas. The RF-9 antagonist we used is potent and selective, displaying the same affinity and antagonist activity at NPFF1R and NPFF2R subtypes [56]. Shortage of selective, with good affinity NPFF1 and NPFF2 antagonists make it difficult to distinguish the NPFF receptor subtype involved in the effect [57]. Nevertheless, previous reports investigating RF-9 showed its ability to block NPFF-induced hypertension, heart rate increase [56], and hypothermia [58]. RF-9 eliminated also heroine-induced hyperalgesia and tolerance [56], and morphine-induced analgesia [58].

Regarding cardiovascular effects, namely elevation of blood pressure and heart rate produced with systemic administration of NPFF, we did not investigate the mechanism of these responses, however our study confirmed earlier reports. The same effects were presented after iv NPFF and attributed to the catecholamine dependent mechanism and excitation of heart NPFF receptors [20]. Intrathecal NPFF evoked also pressor and tachycardia response mediated most probably via spinal NPFF receptors with the share of muscarinic receptors and adrenoceptors [15]. Central injection mediated hypertension [23,56,59] and heart rate control via central structures responsible for the control of the sympathetic tone [20], however icv application evoked tachycardia [56,59], in contrast to bradycardia noted after microinjection into the commissural NTS [23].

In our study intravenously given NPFF, in contrast to the icv route of application, was not effective in blocking cardiovascular responses induced by EM-1. The reason is not known, however the explanation was discussed earlier as a limited possibility of NPFF to cross BBB. Given that the neuropeptide simultaneously blocks opioid apnea and its analgesic effect and has a limited ability to cross the blood–brain barrier, its practical use in reversing respiratory depression is not possible at present. Nevertheless, the creation of a new selective compound that acts on a specific type of NPFF receptor and passes BBB could provide such an opportunity.

In conclusion, our results showed that centrally administered neuropeptide FF plays an important role in the expression of vagally mediated apnea induced with systemic stimulation of μ-opioid receptors. It appeared to be clear that NPFF anti-EM-1 action was due to stimulation of central NPFF receptors. Our finding has uncovered a novel role for NPFF and its receptors and provided a potential new target for reversal of opioid-induced respiratory depression. 

## 4. Materials and Methods

### 4.1. Animals

All animal procedures were approved by the Local Ethical Committee in Warsaw (Poland, permit No. 433/2017, consent granted on 8 November 2017) and performed in accordance with the European Legislation (2010/63/EU). Experiments were performed on adult male Wistar rats (*n* = 59) weighing 262 ± 24 g under urethane (750 mg/kg) and α-chloralose (600 mg/kg; Sigma-Aldrich, Poznań, Poland) anesthesia administered intraperitoneally. The level of unconsciousness was controlled by the observation of the changes in arterial blood pressure induced by the noxious stimuli (pinch paw). Supplementary doses of anesthesia were administered if necessary.

### 4.2. Surgical Procedures

The animals were placed in supine position and its rectal temperature was maintained close to 37–38 °C. Trachea was exposed, dissected below the larynx and supported with a tracheostomy tube through which the animal was breathing room air. Femoral artery for blood pressure monitoring and femoral vein for drug administration were catheterized.

After preliminary surgery (vein and artery catheterization, tracheostomy and securing the bipolar electrodes in the sternal part of the diaphragm) the animal’s head was mounted in the stereotactic apparatus and pneumotachograph head connected to the tracheal tube. Coordinates for right lateral ventricle were adjusted according to the Paxinos and Watson Atlas, 2006 [60] and a 10 μL Hammilton syringe equipped with a stainless steel needle (21 G) was inserted stereotactically to a depth of 3.5 mm in a position 1.4 mm to the right and 0.85 mm caudal to bregma. The proper microinjection site was checked post-mortem via examination of the needle trace leading through the cortex to the lateral ventricle.

### 4.3. Registration and Counting of Parameters

Respiratory signal was registered with a pneumotachograph head connected to the tracheal cannula and linked to a Research Pneumotach System and a computerized recording system (RSS 100 HR, Hans Rudolph Inc., Kansas City, MO, USA) and tidal volume (V_T_), air flow, breathing frequency (F), and minute ventilation (V_E_), time of inspiration (T_I_), and expiratory time (T_E_) were recorded.

The electromyogram of costal diaphragm was recorded from its sternal part with bipolar electrodes. The signal was amplified (1000–5000×) with a NL 104 amplifier (Digitimer), band-pass filtered (50 Hz–50 kHz) and combined with a model AS 101 (Asbit) leaky integrator (time constant = 100 ms).

Arterial blood pressure and heart rate were measured with a BP-2 blood pressure monitor (Columbus Instruments, Columbus, OH, USA) connected to the catheter placed in the femoral artery. The respiratory recordings (tidal volume and air flow), blood pressure, and integrated electromyogram of the diaphragm were registered with an Omnilight 8 M 36 apparatus (Honeywell, Tokyo, Japan).

The values of respiratory pattern parameters, mean arterial blood pressure (MAP), and heart rate (HR) were calculated by averaging the variables measured for five sequential respiratory cycles just prior to drug injection (baseline) and at chosen time points of the post-challenge phase. The maximal and minimal post-EM-1 changes in measured parameters were chosen from the computed time points ranging from the early post-drug phase to 5 min. The duration of the apneic period in expiratory airflow was measured as the time of respiratory arrest. Prolongation of T_E_ was measured as the ratio of maximal T_E_ during post-EM-1 apnea or expiration (T_E_ test) to control expiratory time (T_E_ control), T_E_ test/T_E_ control [41].

### 4.4. Drugs

Both endomorphin-1 (EM-1; Tocris Bio-Techne, Warsaw, Poland) and neuropeptide FF (NPFF; Sigma-Aldrich, Poznań, Poland) were prepared freshly from powder before each experiment. RF9-TCA (Sigma-Aldrich, Poznań, Poland) was dissolved in physiological saline, divided into 5–10 μL aliquots and stored in −80 °C until use.

Drugs were dissolved in physiological saline and administered as follows:

1. First, control group of animals received an intravenous (iv) injection of EM-1 in a dose of 50 μg/kg;
2. Second group was divided on rats that received iv injection of NPFF in doses of 0.1, 0.3, 0.6 and 1.2 mg/kg 2 min prior to the EM-1 challenge;
3. Third group of animals was divided on rats that were administered icv (right cerebral ventricle) with vehicle (saline) and NPFF in doses of 1, 10 and 20 μg per rat before iv EM-1 injection;
4. Fourth group was injected icv with the mixture (per rat) of 20 μg of neuropeptide FF and 20 μg of RF9—neuropeptide FF receptor antagonist, before the intravenous EM-1 challenge.

EM-1 was administered 2 min after iv pretreatment with saline or the NPFF challenge. Intravenous drug volumes did not exceed 0.3 mL. Volumes of icv injections was 5 μL and lasted 5 min, EM-1 was injected intravenously afterwards. Signals were registered as described above.

### 4.5. Statistics

Since not all the data were normally distributed and because of a small group size, statistical analysis was carried out using non-parametric tests. The results are presented as medians with quartile deviations (QDs). A Wilcoxon test was used for comparison within the group; between prechallenge and defined time points after drug challenge. Differences between individual time points and groups were evaluated by the Mann–Whitney *U* test. The data were analyzed with STATISTICA 12 (StatSoft Polska, Poland). In all cases *p* ≤ 0.05 was considered statistically significant.

## Figures and Tables

**Table 1:** Modulation of cardiovascular effects of iv EM-1 by iv administration of NPFF.

		Saline	NPFF 100 μg	NPFF 300 μg	NPFF 600 μg
	*n*	7	6	5	6
**HR** **1/min**	Baseline	473 ± 37	454 ± 19	491 ± 0	505 ± 38
	MIN 0–30 s	396 ± 18 ^*^	397 ± 22 ^*^	450 ± 9 ^*^ ^#^	450 ± 20
	Recovery (1 min)	454 ± 38	438 ± 19	483 ± 2	492 ± 39
**MAP** **mmHg**	Baseline	80 ± 6	83 ± 6	75 ± 1	84 ± 3
	MIN 0–30 s	29 ± 11 ^*^	66 ± 13 ^*#^	47 ± 5 ^*^	59 ± 9 ^*^
	Recovery (1 min)	81 ± 4	84 ± 12	75 ± 3	79 ± 17

Median ± QD; Wilcoxon test: * *p* < 0.05 vs. Baseline; Mann–Whitney test: # *p* < 0.05 vs. control (saline) group.

**Table 2:** Modulation of cardiovascular effects of intravenous EM-1 by icv administration of neuropeptide FF.

		Saline	NPFF 1 μg	NPFF 10 μg	NPFF 20 μg	NPFF 20 μg RF9 20 μg
	*n*	7	5	7	5	6
**HR** **1/min**	Baseline	428 ± 39	410 ± 32	430 ± 31	459 ± 15	413 ± 56
	MIN 0–30 s	387 ± 11 ^*^	394 ± 34 ^*^	398 ± 33	400 ± 5 ^#^	354 ± 33 ^*^
	Recovery (1 min)	412 ± 18	426 ± 12	455 ± 20 ^*^	456 ± 23	432 ± 31
**MAP** **mmHg**	Baseline	81 ± 4	75 ± 15	77 ± 6	76 ± 7	73 ± 7
	MIN 0–30 s	51 ± 2 ^*^	43 ± 3 ^*#^	68 ± 21	66 ± 5	52 ± 16 ^*^
	Recovery (1 min)	81 ± 7	67 ± 15	89 ± 10	85 ± 7 ^*^	73 ± 9

Median ± QD; Wilcoxon test: * *p* < 0.05 vs. Baseline; Mann–Whitney test: # *p* < 0.05 vs. control (saline) group.
